# In-vitro glistening formation in six different foldable hydrophobic intraocular lenses

**DOI:** 10.1186/s12886-021-01879-6

**Published:** 2021-03-08

**Authors:** Tamer Tandogan, Gerd U. Auffarth, Hyeck-Soo Son, Patrick Merz, Chul Young Choi, Ramin Khoramnia

**Affiliations:** 1 The David J. Apple International Laboratory for Ocular Pathology and International Vision Correction Research Centre (IVCRC), Department of Ophthalmology, University of Heidelberg, INF 400, 69120 Heidelberg, Germany; 2 Department of Ophthalmology, Kangbuk Samsung Hospital, Sungkyunkwan University, Seoul, South Korea

## Abstract

**Background:**

Glistenings describe small, refractile microvacuoles that may arise within the intraocular lens (IOL) material and reduce the patients’ quality of vision. Lenses composed of hydrophobic acrylic material are particularly affected by glistening formation. In this study, we compared the tendency of glistening formation in six different types of hydrophobic acrylic intraocular lenses (IOLs).

**Methods:**

We used a well-established accelerated laboratory method to develop glistenings in the following IOLs: Vivinex XY1 (Hoya), AcrySof SN60WF (Alcon), Tecnis ZCB00 (AMO), Avansee PN6A (Kowa), Aktis SP NS-60YG (Nidek), and CT Lucia 601P (Zeiss). IOLs were first immersed in saline at 45 °C for 24 h and then at 37 °C for 2.5 h in a water bath. Microvacuole (MV) density and size (Miyata grading) were documented and calculated using an image analysis program.

**Results:**

The mean glistening density [MV/mm^2^] and mean Miyata grading (in brackets) were: Vivinex: 11.6 ± 5.7 (0), SN60WF: 264.4 ± 110.3 (2.6), Tecnis: 6.0 ± 2.8 (0), Avansee: 2.2 ± 0.7 (0), Aktis: 851.4 ± 59.4 (3+) and CT Lucia: 71.0 ± 71.6 (1).

**Conclusions:**

While all tested IOLs showed glistenings with the accelerated laboratory method, the Aktis and SN60WF showed the highest microvacuole density, followed by the CT Lucia. In comparison, the Vivinex, Tecnis, and Avansee IOLs showed far fewer number of glistenings.

## Introduction

Glistenings have proven to be of significant interest to clinicians owing to their potentially negative impact on patients’ visual function [1–4]. Various studies have suggested that severe glistenings could mildly reduce contrast sensitivity and visual acuity [5–9].

Glistening involves the formation of aqueous-filled microvacuoles (MV) in implanted intraocular lenses (IOLs), and is highly dependent on IOL material; the incidence and severity are reported to be highest amongst IOLs made up of hydrophobic acrylic materials [6, 10, 11]. According to a survey conducted by the American Society of Cataract and Refractive Surgery, foldable hydrophobic acrylic lenses are the most commonly implanted IOLs in the United States [12]. However, while some of these lenses show severe glistening formation [13–20], other hydrophobic acrylic IOLs have been reported to be free of glistenings up to 2 years after implantation [21].

The in-vitro evaluation of glistenings is challenging due to the slow development of microvacuoles in the IOL. Using a laboratory setting, the formation of glistenings may be simulated and accelerated. While not perfectly representative of in-vivo conditions, in-vitro studies are nevertheless considered valuable in providing information about the tendency of a material to form glistenings [7, 13–15]. Different techniques have been proposed to create glistenings in-vitro [13–19]. In this study, we used the method published by Thomes and Callaghan to generate glistenings under laboratory conditions [13] and compared acute glistening formation in six different hydrophobic acrylic IOL models by analyzing the microvacuole density and size according to the Miyata grading system [14].

## Materials and methods

Six models of foldable IOL were analyzed in this comparative trial: the Vivinex XY1 (Hoya), the AcrySof SN60WF (Alcon), the Tecnis ZCB00 (AMO), the Avansee PN6A (Kowa), the Aktis SP NS-60YG (Nidek) and the CT Lucia 601P (Zeiss). For each IOL model, we analyzed five IOLs. All IOLs had + 20 D power and were made of clear (not blue-light filtering) hydrophobic acrylic with an integrated UV filter. Table 1 shows the material composition and manufacturing methods of the IOLs.

**Table 1:** Optic material and manufacturing methods of the intraocular lenses

IOL Model	Optic material composition	Manufacturing Process
Hoya Vivinex™ XY1	Crosslinked copolymer of phenylethyl methacrylate and n-butyl acrylate, fluoroalkyl methacrylate	Lathe-cut
AMO Tecnis® ZCB00	Copolymer of ethyl acrylate, ethyl methacrylate, 2.2,2-trifluorethyl methacrylate, crosslinked with ethylene glycol dimethacrylate	Cryo-lathing
Kowa Avansee™ PN6A	Crosslinked copolymer of 2-phenoxyethyl acrylate and ethyl acrylate	Cast-molding
Alcon AcrySof® SN60WF	Copolymer of phenylethyl acrylate and phenylethyl methacrylate, crosslinked with butanediol diacrylate	Cast-molding
Zeiss CT LUCIA® 601P	Copolymer of butyl acrylate, ethyl methacrylate and N-benzyl-N-isopropylpropenamide Heparin Coated Surface	Lathe-cut
Nidek Aktis SP NS-60YG	Copolymer of n-butyl acrylate, n-butyl methacrylate and phenoxyethyl acrylate	Lathe-cut

We used the accelerated ageing simulation method, as described by Thomes and Callaghan [13], on all IOLs. In brief, the IOLs were placed in flasks that were filled with balanced salt solution. The IOLs were always kept in a wet state during the course of the study. These flasks were placed in a climatic chamber set to 45 ± 1 °C. After 24 h, the IOLs were moved to a 37 °C ± 1 °C water bath, where they remained for another 2.5 h. Samples were analyzed after ageing simulation was completed using a heated stage microscope (MEIJI EMZ-TR8), a CCD camera, a computer, and image analysis software (iSolution). The IOLs were inspected visually via light microscopy. All IOLs were evaluated at the specific temperature of 37 °C. The heated stage enabled maintenance of the IOL at this temperature during imaging. This accounted for maintaining stable microvacuole size and density during inspection.

In each lens, the area of the optical zone with the densest distribution of microvacuoles was selected for comparative analysis. For this purpose, the entire lens was scanned and the region of maximum density (central or paracentral and at the correct focal plane) was imaged. The image analysis program then processed the images. Data from these processed images was used to evaluate microvacuole density (MVs/mm^2^).

Statistical analysis was performed using SPSS (IBM SPSS Statistics, V.22). As the data did not satisfy the normality distribution (Kolmogorov-Smimov test) and equality of variance assumption (Levene test), all data were statistically evaluated using nonparametric (Kruskal-Wallis) tests. A *p*-value of less than 0.05 was recognized as statistically significant.

## Results

Table 2 summarizes the results of all IOLs. All lenses demonstrated glistening formation following the accelerated ageing process, however there were large differences between the various IOL models (*p* < 0.001, Kruskal-Wallis test).

**Table 2:** Overview of the glistening densities and the Miyata grading [14] of all six intraocular lenses

IOL Model	Glistenings/mm^2^ (mean ± standard deviation)	Miyata grade (mean)
Hoya Vivinex™ XY1	11.6 ± 5.7	0
AMO Tecnis® ZCB00	6.0 ± 2.8	0
Kowa Avansee™ PN6A	2.2 ± 0.7	0
Alcon AcrySof® SN60WF	264.4 ± 110.3	2.6
Zeiss CT LUCIA® 601P	71.0 ± 71.6	1
Nidek Aktis SP NS-60YG	851.4 ± 59.4	3+

*IOL* Intraocular lens

**Fig. 1:**
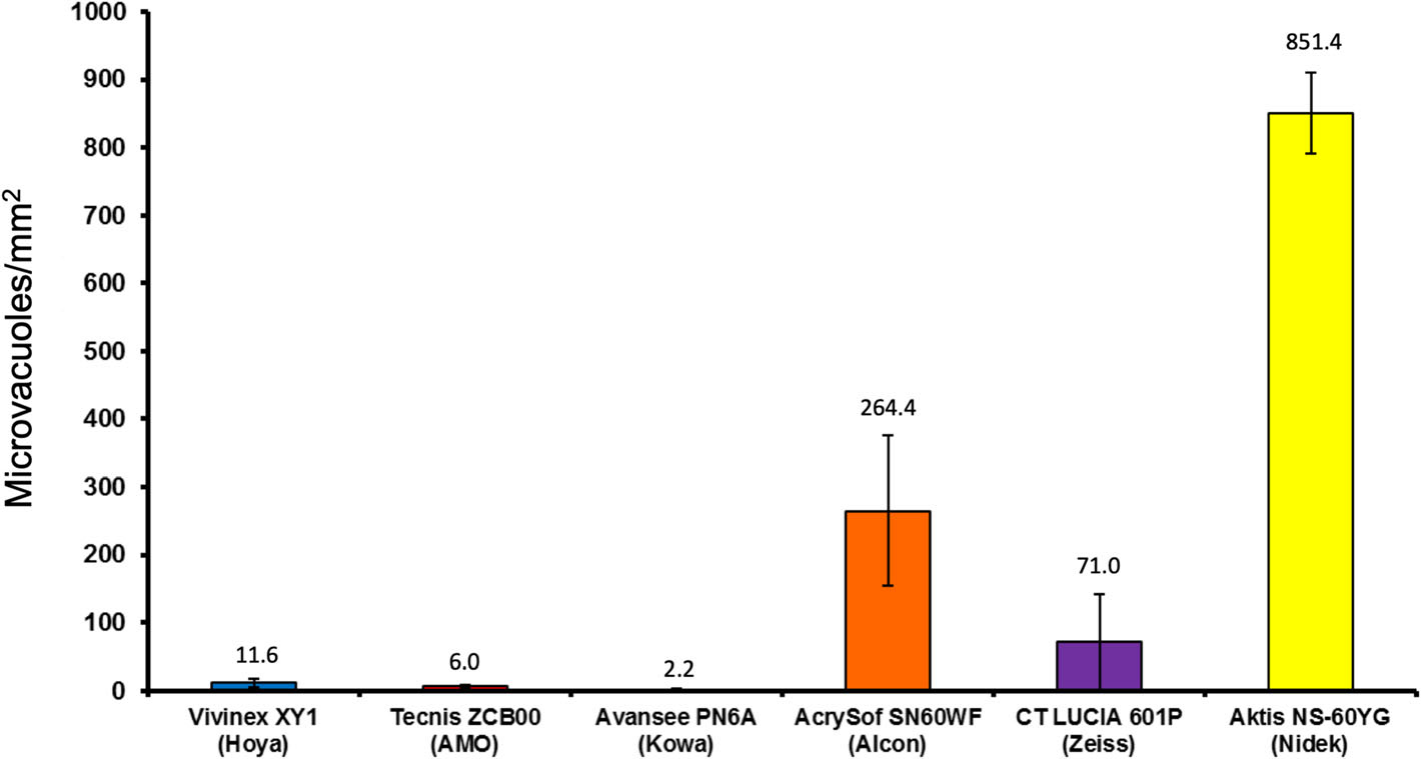
Mean microvacuole density of the six hydrophobic acrylic intraocular lenses

The Nidek IOL showed very large glistening densities. The Alcon IOL had only 31% glistenings compared to the Nidek IOL. The Zeiss IOL demonstrated 27% glistenings compared to the Alcon IOL (8% of the Nidek IOL). The Hoya, AMO and KOWA IOLs showed only a few glistenings at all (Fig. 1).

**Fig. 2:**
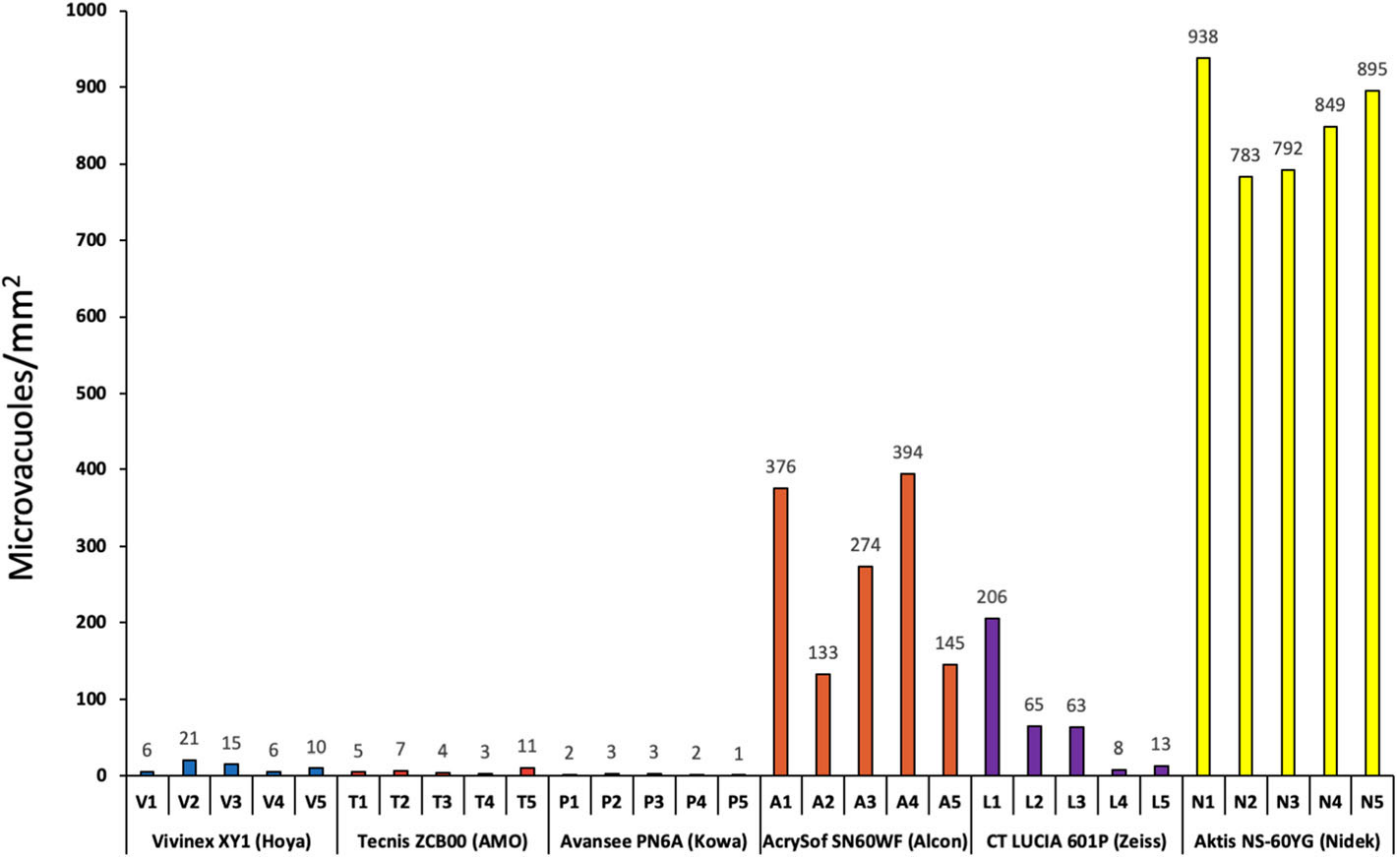
Microvacuole density in each of the six intraocular lens models; five individual lenses per model

The analysis of the individual IOL per model group (Fig. 2) shows, at least in part, large relative intra-model differences for the Alcon and especially for the Zeiss IOLs, while the relative differences between the Nidek IOLs are fewer.

**Fig. 3:**
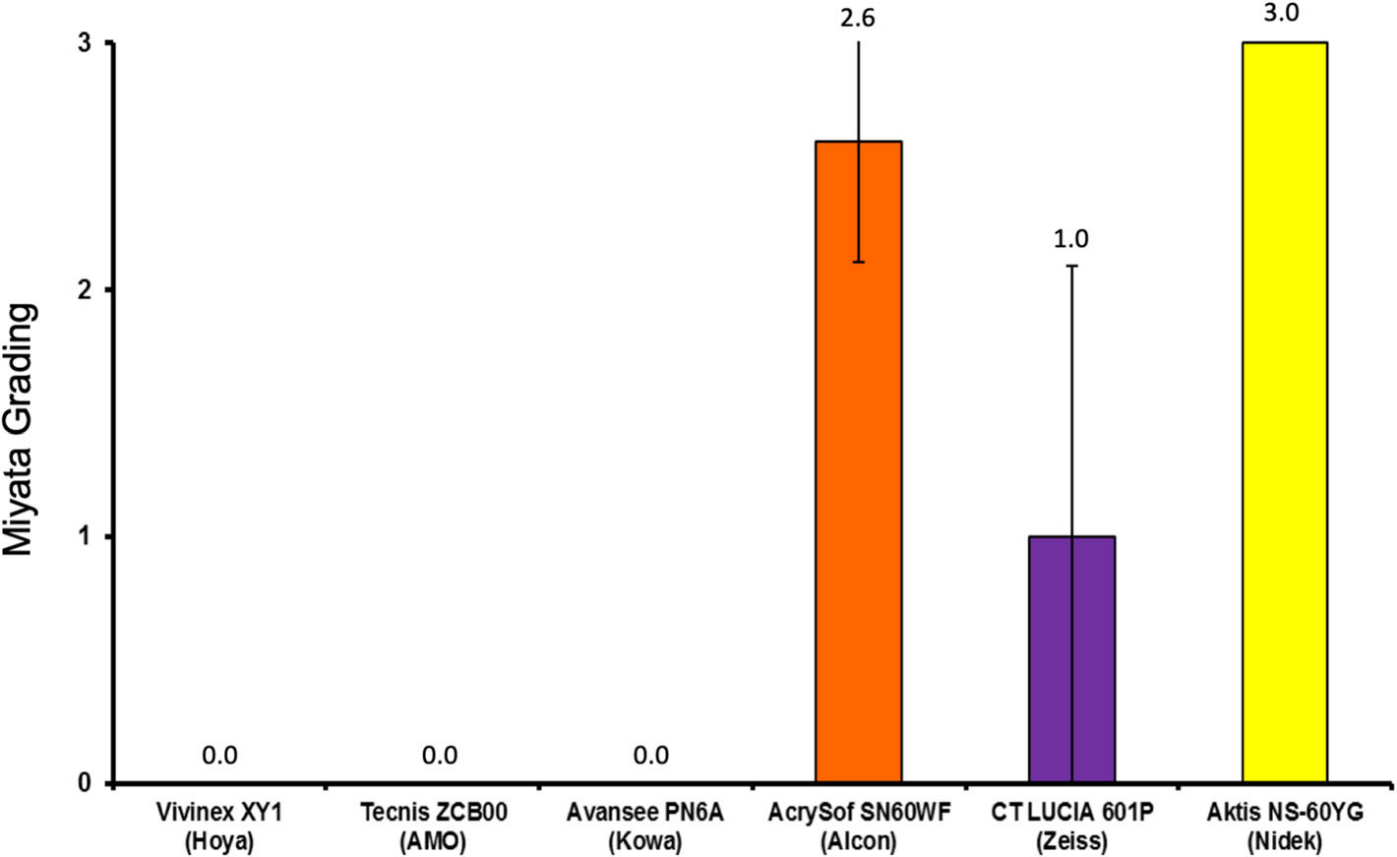
Mean Miyata grading [14] of all six lens models

**Fig. 4:**
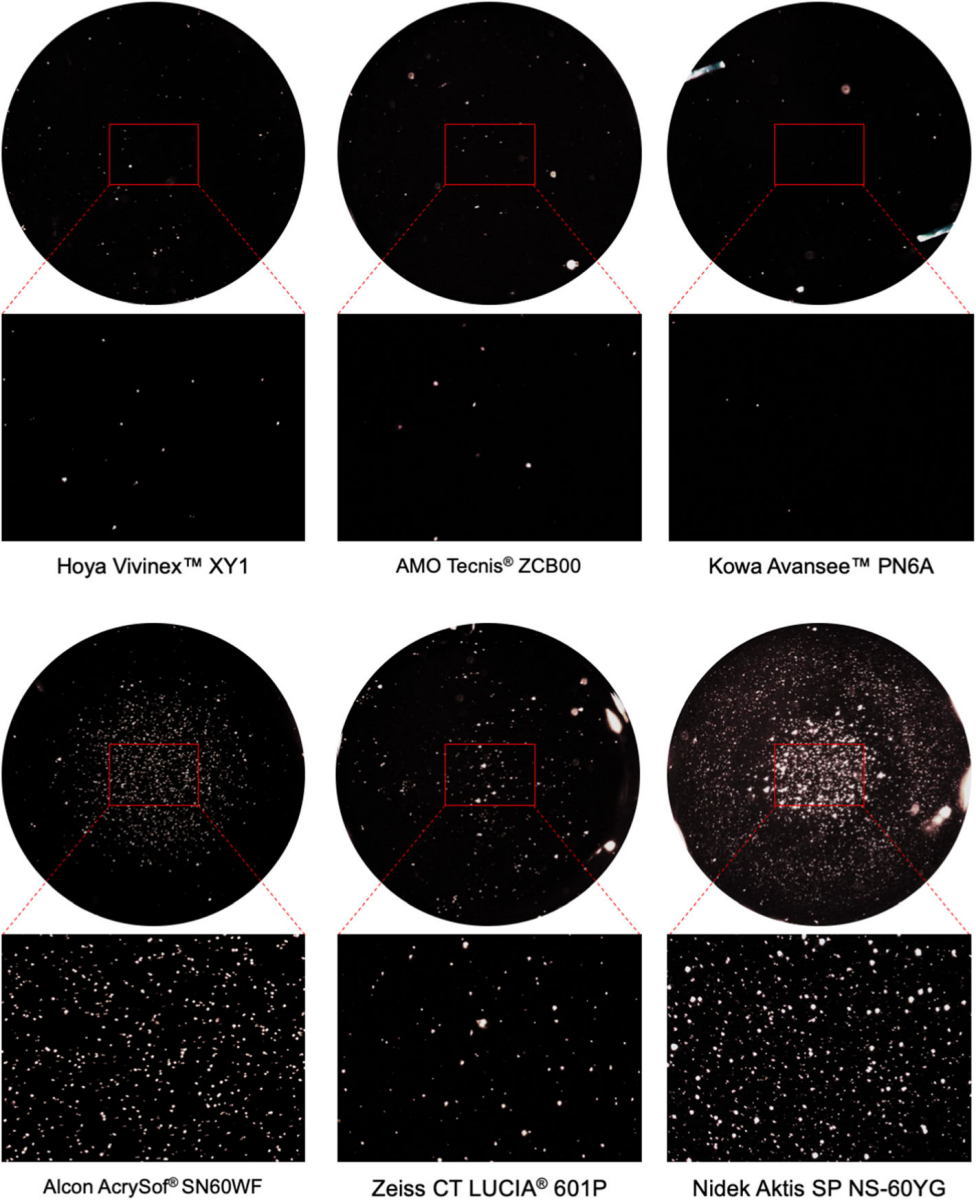
Light microscopic images of all examined intraocular lenses after glistening formation (14x, 90x magnification)

This trend is also reflected in the Miyata grading results (Fig. 3); the Hoya, AMO and KOWA materials show the lowest grading, while the Zeiss lenses are classified slightly higher and the Alcon and Nidek IOLs are graded on the top end of the grading system. Figure 4 shows the microscopic images of IOLs after glistening formation.

## Discussion

The negative impact of glistenings on visual functions such as visual acuity and contrast sensitivity has been suggested by several studies [7, 8, 22, 23]. It is noteworthy, however, that these studies revealed very small deteriorations in visual function, and this too only in cases with severe amounts of glistening formation.

Glistenings are usually considered to be fluid-filled microvacuoles. They form within the IOL matrix under exposition to aqueous environments [13]. As there is a significant difference in the refractive indices of the liquid-filled vacuoles (*n* = 1.33) and the polymer body of the IOL (*n* = 1.55, depending on the IOL material), light is refracted and scattered at the water-polymer border. The vacuoles thus become visible using a slit lamp or under light microscopy [16]. Their formation appears to be most prominent in hydrophobic acrylic IOLs [6, 10, 11].

In our study, glistenings – or microvacuoles – were developed by utilizing an accelerated microvacuole test method on different IOL models [13]. We used temperature changes in an aqueous environment to accelerate the formation of glistenings, performing an optical purity assessment by quantifying density and size of glistenings in those IOLs.

We tested six different hydrophobic IOL models in this laboratory setting, with the results demonstrating that no IOL was completely glistening-free at the end of the accelerated ageing procedure. Even though all six IOL models were hydrophobic acrylic ones, the testing revealed large inter-IOL and also some intra-IOL differences.

The tendency of Alcon AcrySof IOLs to form glistenings under laboratory conditions correlates well with the findings of previously published clinical studies [6, 10, 11]. In the case of the Nidek Aktis model, glistenings were even more prominent within this study. Despite the larger amount of glistenings in the Aktis, the glistening size grading for both the Nidek and Alcon IOLs is almost the same. Overall, the Zeiss Lucia IOL showed smaller and fewer glistenings. Honing into the intra-IOL differences of glistening density in each group demonstrates that the Zeiss IOL shows one extreme value, which increases the resulting mean. It is unclear why this particular lens had a much larger glistening formation than the other specimen.

If one compares the intra-IOL differences of all IOL groups, it becomes apparent that the Alcon, Zeiss and Nidek IOLs show very similar intra-IOL differences. As the average values of glistening density in the Zeiss IOL are much lower than in the Alcon or Nidek IOL, the extreme value causes this larger impact on the means. The other lenses (Hoya Vivinex, AMO Tecnis and KOWA Avansee) showed very few and very small glistenings only, which can be considered clinically irrelevant.

These lower values of glistening formation have been corroborated for the Tecnis material in various studies [24, 25]. There appears to be no previously published data for the other IOLs in this study (Vivinex, Avansee, Lucia and Aktis).

Within the in-vivo aqueous environment of the human eye, certain temperature fluctuations might occur, which are not reflected by our laboratory-based testing methods. The morphological aspects apparent in laboratory testing are usually considered as being exaggerated compared to in-vivo formations. While several studies confirm the suitability of such in-vitro testing methods for clinical assessment, temperature fluctuations might trigger the development of different characteristics of glistenings and their formation compared to the laboratory setting [11, 17, 24]. The rate of temperature fluctuations appears to have an effect on the extent of glistening formation. Furthermore, it remains somewhat unclear whether glistenings produced with such in-vitro laboratory methods form due to the same principle or are of the same kind as glistenings observed in the clinical setting in human patients [13].

Osmolarity of the aqueous around the IOL may play an additional role in glistening formation in the individual patient. This might also be said of certain comorbidities, such as diabetes mellitus, glaucoma, inflammatory conditions or a disturbed blood-aqueous barrier.

Overall, in-vitro analysis as performed in our study does provide an assessment of the tendency of a material to form glistenings. The correlation between in-vitro test results and in-vivo observations, however, remains unclear.

It is important to note that the issue of glistening formation may have been solved by introduction of the novel Clareon material (Alcon), which was shown to be glistenings-free in preclinical in-vitro studies (Auffarth GU, ESCRS 2017). Similarly, Hoya has also developed a new glistening-free material called Vivinex (Auffarth GU, ESCRS 2017).

Whether glistenings may lead to any clinically relevant disturbances of visual function of the pseudophakic visual system and an understanding of the evolution of those disturbances in the late postoperative period remains an issue of debate. Some studies showed that there is no relevant impact of glistenings on vision [10, 26, 27]. Others reported a very limited impact on visual acuity, contrast sensitivity at high spatial frequency or intraocular stray light [5, 22, 28].

Our results offer a comparison between different IOL models with regard to their tendency to form glistenings.

## Data Availability

Authors can confirm that all relevant data are included in the article. The datasets used for analysis are available from the corresponding author on reasonable request.

## References

[1] Khoramnia R, Yildirim TM, Łabuz G, Mayer CS, Auffarth GU. Eintrübung von Intraokularlinsen: Erkenntnisse aus dem Labor und der Klinik [Opacification of intraocular lenses: laboratory and clinical findings]. *Ophthalmologe*. 2020 Nov 13. German. 10.1007/s00347-020-01259-3 PMC8260513 33188443

[2] Kanclerz P, Yildirim TM, Khoramnia R (2021). A review of late intraocular lens opacifications. Curr Opin Ophthalmol.

[3] Kanclerz P, Yildirim TM, Khoramnia R. Microscopic Characteristics of Late Intraocular Lens Opacifications. Arch Pathol Lab Med. 2020. 10.5858/arpa.2019-0626-RA 33091924

[4] Weindler JN, Łabuz G, Yildirim TM, Tandogan T, Khoramnia R, Auffarth GU (2019). The impact of glistenings on the optical quality of a hydrophobic acrylic intraocular lens. J Cataract Refract Surg.

[5] Beiko GH, Grzybowski A (2013). Glistenings in hydrophobic acrylic intraocular lenses do affect visual function. Clin Ophthalmol.

[6] Rønbeck M, Behndig A, Taube M, Koivula A, Kugelberg M (2013). Comparison of glistenings in intraocular lenses with three different materials: 12-year follow-up. Acta Ophthalmol.

[7] Christiansen G, Durcan FJ, Olson RJ, Christiansen K (2001). Glistenings in the AcrySof intraocular lens: pilot study. J Cataract Refract Surg.

[8] Gunenc U, Gunenc U, Oner FH, Tongal S, Ferliel M (2001). Effects on visual function of glistenings and folding marks in AcrySof intraocular lenses. J Cataract Refract Surg.

[9] Matsushima H, Nagata M, Katsuki Y, Ota I, Miyake K, Beiko GH, Grzybowski A (2015). Decreased visual acuity resulting from glistening and sub-surface nano-glistening formation in intraocular lenses: a retrospective analysis of 5 cases. Saudi J Ophthalmol.

[10] Werner L (2010). Glistenings and surface light scattering in intraocular lenses. J Cataract Refract Surg.

[11] Tognetto D, Toto L, Sanguinetti G, Ravalico G (2002). Glistenings in foldable intraocular lenses. J Cataract Refract Surg.

[12] Leaming DV (2004). Practice styles and preferences of ASCRS members--2003 survey. J Cataract Refract Surg.

[13] Thomes BE, Callaghan TA (2013). Evaluation of in vitro glistening formation in hydrophobic acrylic intraocular lenses. Clin Ophthalmol.

[14] Miyata A, Uchida N, Nakajima K, Yaguchi S (2000). Clinical and experimental observation of glistening in acrylic intraocular lenses. Jpn J Ophthalmol.

[15] Kato K, Nishida M, Yamane H, Nakamae K, Tagami Y, Tetsumoto K (2001). Glistening formation in an AcrySof lens initiated by spinodal decomposition of the polymer network by temperature change. J Cataract Refract Surg.

[16] Gregori NZ, Spencer TS, Mamalis N, Olson RJ (2002). In vitro comparison of glistening formation among hydrophobic acrylic intraocular lenses (1). J Cataract Refract Surg.

[17] Kawak K, Hayakawa K, Suzuki T (2012). Simulation of 20-year deterioration of acrylic IOLs using severe accelerated deterioration tests. Tokai J Exp Clin Med.

[18] Miyata A, Yaguchi S (2004). Equilibrium water content and glistenings in acrylic intraocular lenses. J Cataract Refract Surg.

[19] Omar O, Pirayesh A, Mamalis N, Olson RJ (1998). In vitro analysis of AcrySof intraocular lens glistenings in AcryPak and wagon wheel packaging. J Cataract Refract Surg.

[20] Dogru M, Tetsumoto K, Tagami Y, Kato K, Nakamae K (2000). Optical and atomic force microscopy of an explanted AcrySof intraocular lens with glistenings. J Cataract Refract Surg.

[21] Packer M, Rajan M, Ligabue E, Heiner P (2014). Clinical properties of a novel, glistening-free, single-piece, hydrophobic acrylic IOL. Clin Ophthalmol.

[22] Dhaliwal DK, Mamalis N, Olson RJ, Crandall AS, Zimmerman P, Alldredge OC, Durcan FJ, Omar O (1996). Visual significance of glistenings seen in the AcrySof intraocular lens. J Cataract Refract Surg.

[23] Xi L, Liu Y, Zhao F, Chen C, Cheng B (2014). Analysis of glistenings in hydrophobic acrylic intraocular lenses on visual performance. Int J Ophthalmol.

[24] Kahraman G, Amon M, Ferdinaro C, Nigl K, Walch M (2015). Intraindividual comparative analysis of capsule opacification after implantation of 2 single-piece hydrophobic acrylic intraocular lenses models: three-year follow-up. J Cataract Refract Surg.

[25] Nagata M, Matsushima H, Mukai K, Terauchi W, Senoo T, Wada H, Yoshida S (2010). Clinical evaluation of the transparency of hydrophobic acrylic intraocular lens optics. J Cataract Refract Surg.

[26] Wilkins E, Olson RJ (2001). Glistenings with long-term follow-up of the Surgidev B20/20 polymethylmethacrylate intraocular lens. Am J Ophthalmol.

[27] Colin J, Orignac I (2011). Glistenings on intraocular lenses in healthy eyes: effects and associations. J Refract Surg.

[28] Waite A, Faulkner N, Olson RJ (2007). Glistenings in the single-piece, hydrophobic, acrylic intraocular lenses. Am J Ophthalmol.

